# Solubility of Sulfamerazine in Acetonitrile + Ethanol Cosolvent Mixtures: Thermodynamics and Modeling

**DOI:** 10.3390/molecules29225294

**Published:** 2024-11-09

**Authors:** Claudia Patricia Ortiz, Diego Ivan Caviedes-Rubio, Fleming Martinez, Daniel Ricardo Delgado

**Affiliations:** 1 Programa de Administración en Seguridad y Salud en el Trabajo, Grupo de Investigación en Seguridad y Salud en el Trabajo, Corporación Universitaria Minuto de Dios-UNIMINUTO, Neiva 410001, Huila, Colombia; claudia.ortiz.de@uniminuto.edu.co; 2 Programa de Ingeniería Civil, Grupo de Investigación de Ingenierías UCC-Neiva, Facultad de Ingeniería, Universidad Cooperativa de Colombia, Sede Neiva, Calle 11 No. 1-51, Neiva 410001, Huila, Colombia; diego.caviedesr@campusucc.edu.co; 3 Grupo de Investigaciones Farmacéutico-Fisicoquímicas, Departamento de Farmacia, Facultad de Ciencias, Universidad Nacional de Colombia, Sede Bogotá, Carrera 30 No. 45-03, Bogotá 110321, Cundinamarca, Colombia; fmartinezr@unal.edu.co

**Keywords:** sulfamerazine, solubility, infections, shake-flask method, thermodynamic properties, van’t Hoff, Yalkowsky–Roseman, binary mixtures

## Abstract

Sulfamerazine (SMR) is a drug used as an antibacterial agent in the treatment of some pathologies, such as bronchitis, prostatitis and urinary tract infections. Although this drug was developed in 1945 and, due to its toxicity, was partially displaced by penicillin, due to the current problem of bacterial resistance, compounds such as SMR have regained validity. In this context, the thermodynamic study of SMR in cosolvent mixtures of acetonitrile (MeCN) + ethanol (EtOH) at nine temperatures (278.15–318.15 K) is presented. The solubility of SMR was determined by UV–Vis spectrophotometry, following the guidelines of the shake-flask method. The solubility process was endothermic in all cases; thus, the minimum solubility was reached in pure EtOH at 278.15 K, and the maximum solubility was reached in pure MeCN at 318.15 K. Both the solution process and the mixing process were entropy-driven. On the other hand, the solubility data were modeled by using the van’t Hoff–Yalkowsky–Roseman model, obtaining an overall average relative deviation of 3.9%. In general terms, it can be concluded that the solution process of SMR in {MeCN (1) + EtOH (2)} mixtures is thermodependent, favored by the entropy of the solution and mixture; additionally, the van’t Hoff–Yalkowsky–Roseman model allows very good approximations to be obtained and is a simple model that starts from only four experimental data.

## 1. Introduction

**Figure 1:**
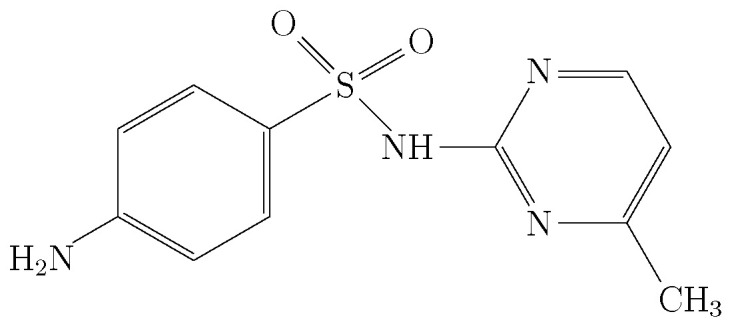
Molecular structure of sulfamerazine [5].

Sulfamerazine (IUPAC name: 4-amino-*N*-(4-methylpyrimidin-2-yl), benzenesulfonamide; molecular formula: C_11_H_12_N_4_O_2_S; CAS: 127-79-7 (Figure 1)) is a bacteriostatic drug which acts as a competitive antagonist of para-aminobenzoic acid (PABA) by preventing the synthesis of folic acid, a precursor of bacterial nucleic acid synthesis. SMR was first synthesized in 1943 by Schmidt et al. [1]. This new drug showed better properties than sulfadiazine (SD) because it is absorbed faster, and in relation to sulfamethazine (SMT), SMR is eliminated more slowly, so SMR reaches better blood concentrations, as well as posing lower risk of nephrological damage compared with SD [2]. Although, due to their toxicity, sulfonamides were replaced by penicillins [3], due to the emergence of bacterial resistance, the use of sulfonamides in the treatment of bacterial infections has been resumed [4].

Currently, SMR is used in human therapy in the treatment of infections, especially urinary infections, as well as being widely used in veterinary medicine [6], which has led to significant environmental problems, to the point of this compound being classified as an emerging contaminant by the NORMAN network [7].

Solubility is one of the most important physicochemical properties and is directly related to pharmacological processes, such as the pharmacokinetics and pharmacodynamics of the drug [8], and pharmacotechnical processes, such as preformulation, formulation, quality analysis, crystallization and purification, among others [9,10]; it is also related to environmental processes such as bioremediation and environmental impact assessment [11,12].

Against this background, cosolvency is a technique that consists in mixing miscible solvents together to improve the solubility of a drug [13,14], in addition to providing information that allows for the understanding of possible molecular interactions associated with the development of biological and industrial processes [15].

Two solvents of pharmaceutical relevance are MeCN and EtOH. MeCN is widely used in high-performance liquid chromatography and other analytical quantification techniques [16]; on the other hand, EtOH is one of the most widely used organic solvents in the development of pharmaceutical forms, being used in almost all stages of purification, preformulation, formulation and development of pharmaceutical dosage forms [13,14,15,17]. Therefore, the investigation of drugs in these two solvents offers important information that has the potential to positively impact the area of pharmaceutical sciences.

The present research study aims to further develop the evaluation of the solubility of structurally related drugs such as sulfadiazine (SD) [18,19,20], sulfamerazine (SMR) [21,22] and sulfamethazine (SMT) [23] in cosolvent mixtures of industrial interest with structurally related solvents such as the linear alcohols methanol, ethanol and propanol. In this context, in addition to generating useful information for the industry, we also seek to elucidate the possible molecular interactions that govern the solubility of these drugs. A complete data set that systematically relates structural changes allows for the development of models that explain solubility behavior in a more rational way; thus, the trend of SMR solubility as a function of temperature and cosolvent composition, the thermodynamics of the solution and mixing process, enthalpy–entropy compensation and the van’t Hoff–Yalkowsky–Roseman solubility model are presented.

## 2. Results

### Experimental Solubility of Sulfamerazine ($x_{3}$) in Acetonitrile (1) + Ethanol (2) Cosolvent Mixtures at 9 Temperatures (278.15–318.15 K)

**Figure 2:**
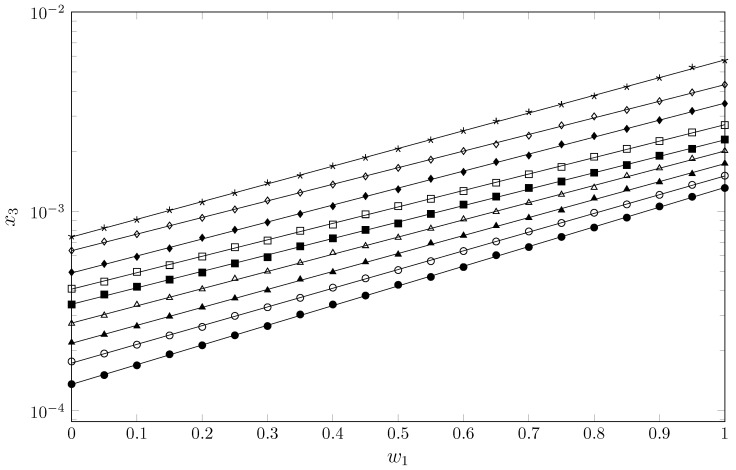
Solubility of SMR (3) ($x_{3}$) as function of mass fraction of MeCN in {MeCN (1) + EtOH (2)} mixtures at different temperatures. •: 278.15 K; ∘: 283.15 K; ▲: 288.15 K; ∆: 293.15 K; ■: 298.15 K; □: 303.15 K; ⧫: 308.15 K; ◊: 313.15 K; ✶: 318.15 K.

Table 1 (Figure 2) shows the experimental solubility of SMR in cosolvent mixtures of MeCN + EtOH. In all cases, the solubility increases with the temperature, indicating an endothermic process; furthermore, as the concentration of MeCN in the cosolvent mixture increases, the solubility of SMR increases, indicating a cosolvent effect of MeCN (20.0 MPa^1/2^ [24]) and an antisolvent effect of EtOH (26.5 MPa^1/2^ [24]). Theoretically, one would expect the opposite behavior, i.e., higher solubility in cosolvent systems or pure solvents with polarities similar to SMR (28.10 MPa^1/2^ [25]) [26,27]. This behavior was also present in studies reported by Cárdenas-Torres et al. and Ortiz et al. in cosolvent systems of acetonitrile + methanol [22] (29.6 MPa^1/2^ [24]) and acetonitrile + 1-propanol [21] (24.5 MPa^1/2^ [24]), where the maximum solubility of SMR is higher in MeCN and lower in alcohols, which have polarities closer to that of SMR. When evaluating the solubility of SMR as a function of the α-scale of solvent hydrogen-bond donor (HBD) acidities [28], there is a relationship between the acidic character of the solvent and the solubility of SMR; thus, in media with a basic character, such as MeCN (α = 0.29 ± 0.06 [28]), SMR achieves higher solubility than in more acidic media, such as alcohols (MeOH (α = 0.990 ± 0.014 [28]), EtOH (α = 0.850 ± 0.022 [28]) and PrOH (α = 0.766 ± 0.015 [28])).

**Figure 3:**
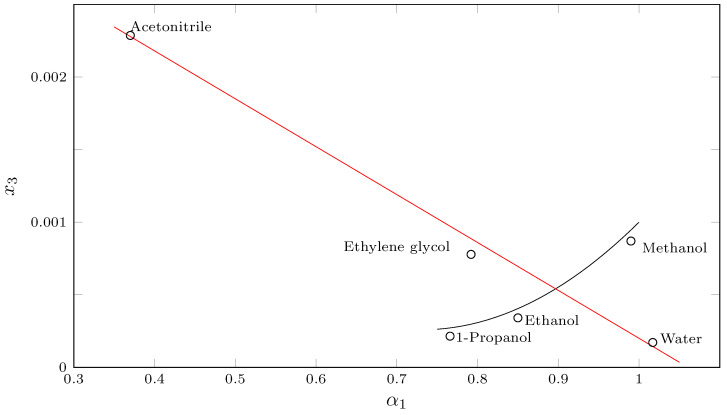
Solubility of SMR (3) ($x_{3}$) as function of α-scale of solvent HBD acidities.

By correlating some SMR solubility data in pure solvents such as water (W) [25,29], acetonitrile [29], ethylene glycol (EG) [30], methanol (MeOH) [22], ethanol [25] and propanol (PrOH) [21] (Figure 3), a quasi-linear relationship is observed between SMR solubility and the acidity parameter (α) of linear, structurally related alcohols (MeOH, EtOH and PrOH). In this case, the increase in solubility could be related to the polarity of the alcohol, since the maximum solubility is reached in MeOH (29.6 MPa^1/2^ [24]); however, when observing the behavior of SMR solubility as a function of α in solvents other than structurally related alcohols (W, EG and MeCN), a linear relationship (r^2^ = 0.998) between α and SMR solubility is observed (Figure 3).

**Figure 4:**
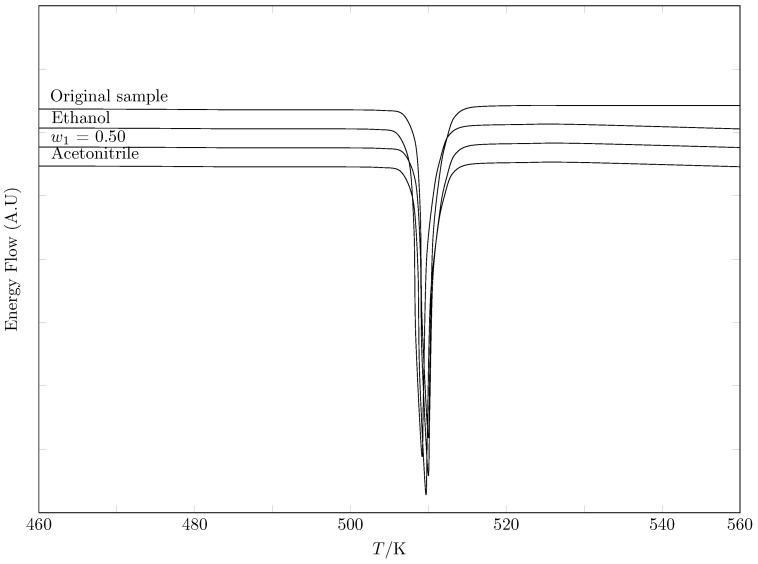
DSC thermograms of SMR.

One factor that can modify the solubility of a drug is the change in the crystalline structure, i.e., possible polymorphic changes [31,32,33]; therefore, it is important to establish whether changes in temperature and cosolvent composition induce polymorphic changes that lead to changes in solubility. Thus, the equilibrium solid phase of SMR in pure MeCN, pure EtOH and the $w_{0.5}$ mixture was evaluated by Differential Scanning Calorimetry (DSC), comparing the results with the DSC of the commercial sample. According to the results shown in Table 2 and Figure 4, there were no polymorphic changes that could alter SMR solubility.

## 3. Thermodynamic Functions

From the solubility data of SMR (3) in cosolvent mixtures {MeCN (1) + EtOH (2)}, the dissolution thermodynamic functions are calculated according to the Gibbs–van’t Hoff–Krug model [40,41], according to expressions (1)–(5).

(1) $$\Delta_{\mathrm{soln}}H^{\circ }=-R{\left(\frac{\partial \ln x_{3}}{\partial \left(T^{-1}-T_{\mathrm{hm}}^{-1}\right)}\right)}_{p}=-R\cdot m$$

(2) $$\Delta_{\mathrm{soln}}G^{\circ }=-RT_{\mathrm{hm}}\cdot \mathrm{intercept}$$

(3) $$\Delta_{\mathrm{soln}}S^{\circ }=\left(\Delta_{\mathrm{soln}}H^{\circ }-\Delta_{\mathrm{soln}}G^{\circ }\right)T_{\mathrm{hm}}^{-1}$$

(4) $$\zeta_{H}=|\Delta_{\mathrm{soln}}H^{\circ }|(|T\Delta_{\mathrm{soln}}S^{\circ }\left|+\right|\Delta_{\mathrm{soln}}H^{\circ }{\left|\right)}^{-1}$$

(5) $$\zeta_{TS}=1-\zeta_{H}$$

where $\Delta_{\mathrm{soln}}H^{\circ }$ (in kJ·mol^−1^), $\Delta_{\mathrm{soln}}G^{\circ }$ (en kJ·mol^−1^) and $\Delta_{\mathrm{soln}}S^{\circ }$ (in ${\mathrm{kJ\cdot mol}}^{-1}\cdot T_{\mathrm{hm}}^{-1}$) are the thermodynamic functions: enthalpy, Gibbs energy and entropy of solution. *T* is the study temperature (in K), $T_{\mathrm{hm}}$ is the harmonic temperature (in K), *R* is the gas constant (kJ·mol^−1^·K^−1^), and $\zeta_{H}$ and $\zeta_{TS}$ are the enthalpic and entropic contributions to the solution process.

The harmonic temperature is calculated as follows:

(6) $$T_{\mathrm{hm}}=\frac{n}{\sum_{i=1}^{n}\frac{1}{T_{i}}}$$

where *n* is the number of study temperatures (in this case, n= 9).

**Figure 5:**
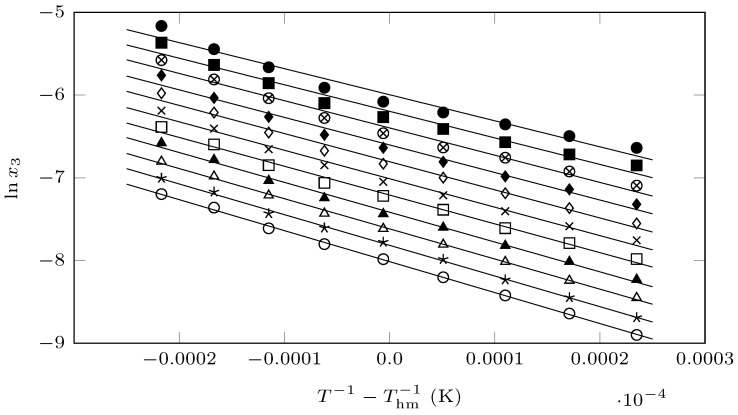
van’t Hoff plot of the mole fraction solubility of SMR ($x_{3}$) in different {MeCN (1) + EtOH (1)} mixture compositions. ∘: neat EtOH; ✶: $w_{0.1}$; ∆: $w_{0.2}$; ▲: $w_{0.3}$; ☐: $w_{0.4}$; x: $w_{0.5}$; *◊*: $w_{0.6}$; ⧫: $w_{0.7}$; ⊗: $w_{0.8}$; ■: $w_{0.9}$; ●: neat MeCN.

From the van’t Hoff–Krug equation (Figure 5),

(7) $$\ln x_{3}=a+m\left(\frac{1}{T}-\frac{1}{T_{\mathrm{hm}}}\right)$$

The enthalpy and Gibbs energy of the solution are calculated, according to Equations (1) and (2), using the values of *m* and *a* (intercept) of Equation (7).

Table 3 shows the dissolution thermodynamic functions $\Delta_{\mathrm{soln}}G^{\circ }$ decreases from pure EtOH to pure MeCN, indicating that the addition of MeCN to SMR favors the solubility of SMR. In regards to $\Delta_{\mathrm{soln}}H^{\circ }$, it is positive in all cases, indicating an endothermic process, as is $\Delta_{\mathrm{soln}}G^{\circ }$. $\Delta_{\mathrm{soln}}G^{\circ }$ decreases from pure EtOH to pure MeCN, presenting a more favorable environment for the solution process. $T\Delta_{\mathrm{soln}}S^{\circ }$ is positive in all cases, indicating an entropic driving of the solution process. When evaluating the contribution of enthalpy and entropy to the value of the Gibbs energy of the solution, the enthalpy of the solution contributes between 69 and 72% in all cases.

**Table 3:** Thermodynamic functions of SMR solution process (3) in {MeCN (1) + EtOH (2)} cosolvent mixtures at $T_{hm}=297.6$ K.

$w_{1}$ ^a^	$\Delta_{\mathrm{soln}}G^{\circ }$	$\Delta_{\mathrm{soln}}H^{\circ }$	$\Delta_{\mathrm{soln}}S^{\circ }$	$T_{\mathrm{hm}}\Delta_{\mathrm{soln}}S^{\circ }$	$\zeta_{H}$ ^b^	$\zeta_{TS}$ ^b^
	(kJ·mol^−1^)	(kJ·mol^−1^**)**	(J·mol^−1^·K^−1^	(kJ·mol^−1^)		
0.00	19.83 ± 0.13	31.15 ± 0.21	38.0 ± 0.4	11.32 ± 0.11	0.733	0.267
0.05	19.6 ± 0.3	31.2 ± 0.29	39.0 ± 0.8	11.61 ± 0.23	0.729	0.271
0.10	19.3 ± 0.4	30.79 ± 0.3	38.5 ± 0.8	11.45 ± 0.25	0.729	0.271
0.15	19.09 ± 0.27	30.44 ± 0.31	38.1 ± 0.7	11.35 ± 0.2	0.728	0.272
0.20	18.85 ± 0.32	30.35 ± 0.28	38.7 ± 0.8	11.5 ± 0.22	0.725	0.275
0.25	18.58 ± 0.33	29.95 ± 0.31	38.2 ± 0.8	11.37 ± 0.23	0.725	0.275
0.30	18.35 ± 0.33	30.0 ± 0.4	39.1 ± 0.9	11.62 ± 0.27	0.721	0.279
0.35	18.08 ± 0.28	29.3 ± 0.4	37.6 ± 0.8	11.19 ± 0.23	0.723	0.277
0.40	17.84 ± 0.26	28.9 ± 0.5	37.3 ± 0.8	11.1 ± 0.25	0.723	0.277
0.45	17.58 ± 0.29	28.9 ± 0.4	38.0 ± 0.9	11.3 ± 0.26	0.719	0.281
0.50	17.35 ± 0.2	28.6 ± 0.5	37.7 ± 0.8	11.23 ± 0.25	0.718	0.282
0.55	17.09 ± 0.27	28.6 ± 0.5	38.6 ± 0.9	11.49 ± 0.28	0.713	0.287
0.60	16.84 ± 0.28	28.3 ± 0.6	38.4 ± 1.0	11.43 ± 0.3	0.712	0.288
0.65	16.58 ± 0.26	27.8 ± 0.7	37.7 ± 1.1	11.22 ± 0.32	0.713	0.287
0.70	16.34 ± 0.26	27.7 ± 0.7	38.1 ± 1.1	11.34 ± 0.34	0.709	0.291
0.75	16.09 ± 0.25	27.7 ± 0.8	39.0 ± 1.2	11.6 ± 0.4	0.705	0.295
0.80	15.83 ± 0.24	27.4 ± 0.8	38.8 ± 1.2	11.5 ± 0.4	0.703	0.297
0.85	15.58 ± 0.23	26.9 ± 0.8	38.0 ± 1.3	11.3 ± 0.4	0.704	0.296
0.90	15.33 ± 0.26	26.7 ± 0.9	38.1 ± 1.4	11.3 ± 0.4	0.702	0.298
0.95	15.07 ± 0.21	26.7 ± 1.0	39.1 ± 1.5	11.6 ± 0.5	0.697	0.303
1.00	14.8 ± 0.45	26.2 ± 0.9	38.1 ± 1.4	11.3 ± 0.4	0.698	0.302

^a^ $w_{1}$ is the mass fraction of acetonitrile (1) in the {acetonitrile (1) + ethanol (2)} mixtures free of SMR (3). ^b^ $\zeta_{H}$ and $\zeta_{TS}$ are the relative contributions by enthalpy and entropy to the apparent Gibbs energy of dissolution.

### 3.1. Thermodynamic Functions of Mixing

**Figure 6:**
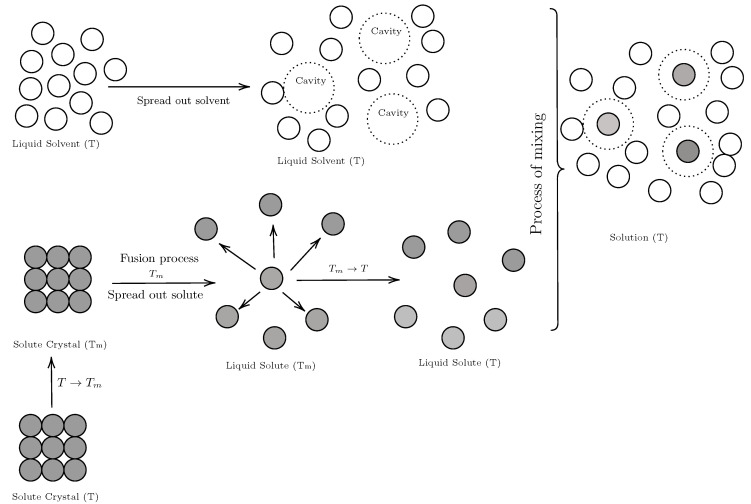
Diagram of hypothetical mixing process (solution formation) [43].

In general, the solution process can be described by three sub-processes [26] (Figure 6):

1. Drug fusion process: In a hypothetical process, the drug changes phase, transforming into a super-cooled liquid. Technically, this process requires energy supply, which is why it is unfavorable for the solution process.
2. Cavity formation: Although the solvent does not present a phase change, the solvent molecules must disintegrate, forming a cavity to house the solute molecule; this process also requires energy investment (endothermic process) and is therefore unfavorable for the solution process [42].
3. Mixing process: Once the drug is in a liquid state and the cavity has been formed in the solvent, the solute molecule is housed in the solvent cavity, forming the solution. This process is exothermic, which favors the solution process.

Mathematically, the solution process can be described as

(8) $$\Delta_{\mathrm{Sol}}f^{o}=\Delta_{\mathrm{mix}}f^{o}+\Delta_{\mathrm{fus}}f^{T_{\mathrm{hm}}}$$

Thus, the thermodynamic mixing functions are calculated as follows:

(9) $$\Delta_{\mathrm{mix}}f^{o}=\Delta_{\mathrm{soln}}f^{o}-\Delta_{\mathrm{fus}}f^{T_{\mathrm{hm}}}$$

where *f* represents the Gibbs energy, enthalpy or entropy of mixing and $f_{\mathrm{fus}}$ represent the thermodynamic functions of the fusion of SMR (3) and its cooling to the harmonic mean temperature, 297.6 K. As it has been described previously in the literature, in this research study, the $\Delta_{\mathrm{soln}}f^{o}$ values for the ideal solution processes were used instead of $\Delta_{f}f^{297.6}$.

Table 4 shows thermodynamic mixing functions, the Gibbs energy of mixing decreases from pure EtOH to pure MeCN, indicating that the mixing process is favored by the addition of MeCN to the system; on the other hand, when evaluating the enthalpy of mixing, this, like the Gibbs energy, decreases from pure EtOH to pure MeCN. This may be related to a lower energy investment in cavity formation in mixtures rich in MeCN and pure MeCN, which may be related to the type of solvent–solvent bonds; thus, EtOH-EtOH interactions may involve H bonds, which are much stronger than MeCN-MeCN bonds, due to the fact that MeCN is an aprotic solvent [44].

The entropy of mixing favors the mixing process, except for $w_{0.35}$ and $w_{0.40}$, whose values are very low, so it is clear that the greatest energy contribution corresponds to the enthalpy of mixing. Moreover, no trend is observed, as the values fluctuate between −0.4±0.9 and 1.4±1.5 ($J\cdot {\mathrm{mol}}^{-1}$·K^−1^).

### 3.2. Enthalpy–Entropy Compensation (EEC) Analysis

The increase in enthalpy due to the effect of the non-covalent solute–solvent interaction is compensated by a simultaneous decrease in entropy, possibly due to greater restriction of the rotational and translational motion of the solute molecule when interacting with the molecules of the solvent(s) [45], due to a thermodynamic effect in response to a perturbation of the equilibrium state [46].

According to Bustamante et al., [47] a graphical method for evaluating EEC consists in plotting the relationship between $\Delta_{\mathrm{soln}}H^{\circ }$ and $\Delta_{\mathrm{soln}}G^{\circ }$.

Thus, according to this graph, enthalpy-driven processes have positive slopes, while entropy-driven processes have negative slopes.

**Figure 7:**
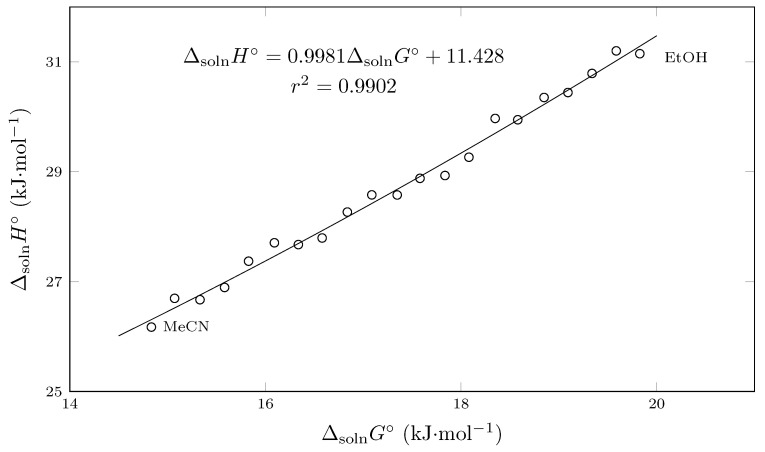
Enthalpy–entropy compensation plot for solubility of SMR (3) in MeCN (1) + EtOH (2) mixtures at $T_{\mathrm{hm}}$ = 297.6 K.

According to Figure 7, it can be observed that the process is conducted by enthalpy, since the slope of the equation describing the behavior is positive. In this context, it is also observed that the relationship is linear, which, according to Chodera and Mobley, is an indicator of a strongly compensated process [48].

### 3.3. Computational Validation

One of the most versatile mathematical models for calculating the solubility of a drug in a cosolvent mixture over a wide range of temperatures and cosolvent compositions is the van’t Hoff–Yalkowsky–Roseman model [49,50].

This model is a combination of two simple equations: the log-linear equation (Equation (10)) proposed by Yalkowsky and Roseman [9,51] and the van’t Hoff equation (Equation (11)) [52].

(10) $$\ln x_{3,1-2}^{C}=w_{1}\ln x_{3,1}+w_{2}\ln x_{3,2}$$

(11) $$\ln x_{3,i}=a_{i}+\frac{m_{i}}{T}$$

Thus, the model equation is presented as

(12) $$\ln x_{3,1-2}=w_{1}\left(a_{1}+\frac{m_{1}}{T}\right)+w_{2}\left(a_{2}+\frac{m_{2}}{T}\right)$$

where $x_{3,1-2}^{C}$ is the solubility of 3 (in this case, SMR) calculated in pure solvents (in this case, MeCN (1) or EtOH (2)) or cosolvent mixture ({MeCN (1) + EtOH (2)}), $x_{3,i}$ is the solubility of 3 in pure solvent (where *i* is MeCN or EtOH), *a* and *b* are the parameters of the van’t Hoff equation and *T* is the temperature in K.

Parameters $a_{1}$, $b_{1}$, $a_{2}$ and $b_{2}$ were calculated from the SMR solubility data in pure MeCN ($x_{3,1-278.15K}=1.36\cdot 10^{-4}$, $x_{3,1-318.15K}=7.49\cdot 10^{-4}$) and EtOH ($x_{3,2-278.15K}=1.31\cdot 10^{-3}$, $x_{3,2-318.15K}=5.72\cdot 10^{-3}$) at 278.15 and 318.15 K, respectively, i.e., four experimental data were used.

Thus, Equation (12) is rewritten as

(13) $$\ln x_{3,1-2}=w_{1}\left(5.086-\frac{3260.9}{T}\right)+w_{2}\left(4.664-\frac{3773.8}{T}\right)$$

To evaluate the model, the experimental and calculated data were correlated by calculating the mean relative deviation (MRD).

(14) $$MRD=\frac{100\sum \left\{\left(x_{3}^{E}-x_{3}^{C}\right)\cdot {\left(x_{3}^{E}\right)}^{-1}\right\}}{N}$$

where $x_{3}^{E}$ is the experimental solubility of SMR.

**Figure 8:**
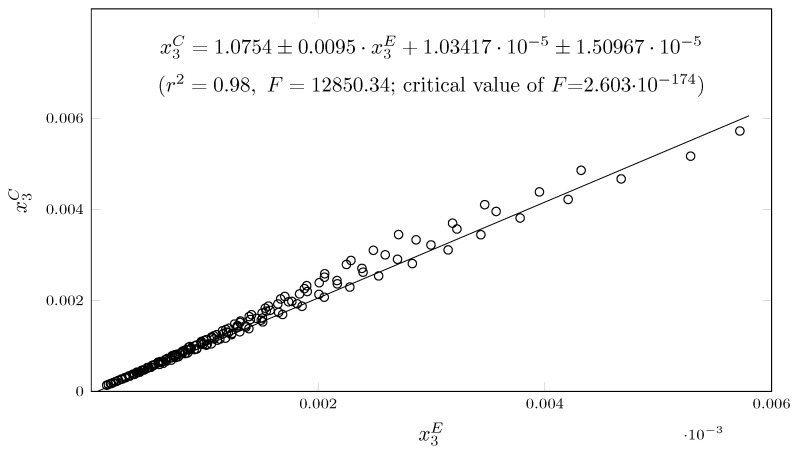
Experimental solubility data versus predicted solubility data for 189 studied solubility data points of SMR in {MeCN (1) + EeOH (2)} mixtures.

When graphing the experimental data vs. the data calculated with the model (Figure 8), an $r^{2}$ of 0.98 is obtained. In addition, the MRD value is 6.13%, indicating a very good correlation of the data.

## 4. Materials and Methods

### 4.1. Reagents

All the reagents used in the research study are reported in Table 5. Some relevant information regarding the quality of each of the reagents is specified.

### 4.2. Solubility Determination

As in other studies published by our group, the solubility of pyrazinamide was determined by using the shake-flask method proposed by Higuchi and Connors [53,54,55]; the method is described in detail in some open-access publications [21].

Overall, the method consists of 5 steps, as follows:

1. Saturation of the solvent: In an amber-colored bottle, 5.0 mL of solvent is added; then, SMR is added with vigorous stirring until a saturated solution is obtained (this process is verified by measuring the concentration of the drug until a constant concentration is obtained).
2. Thermodynamic equilibrium: To ensure solvent saturation, the samples remain for 36 h at constant temperature (at each of the study temperatures) in a recirculation bath (Medingen K-22/T100; Medingen, Germany) to ensure thermodynamic equilibrium. In all cases, a sufficient amount of SMR is added to generate an equilibrium between the saturated solution and a quantity of undissolved solid drug (usually remaining at the bottom of the flask).
3. Filtration: To ensure that no undissolved solids are taken up at the time of quantification, the samples are filtered through 0.45 μm membranes (Swinnex-13; Millipore Corp., Burlington, MA, USA).
4. Quantification: The method used is UV–Vis spectrometry; thus, the wavelength of maximum absorbance of SMR (268 nm ($\lambda_{\max}$)) is determined and a calibration curve is designed in the range of compliance with the Lambert–Beer law (UV–Vis EMC-11 UV spectrophotometer; Duisburg, Germany).
5. Evaluation of the solid phase: To evaluate possible polymorphic changes or decomposition of SMR, the solid phases in equilibrium with the saturated solutions are analyzed by DSC.

### 4.3. Calorimetric Study

The enthalpy and melting temperature of four samples of SMR were determined by DSC (DSC 204 F1; Phoenix, Dresden, Germany). The equipment was calibrated by using indium and tin as standards, and an empty sealed pan was used as reference. A mass of approximately 10.0 mg of each sample was deposited in an aluminum crucible and placed in the calorimeter under a nitrogen flow of 10 mL·min^−1^. A heating cycle to increase the temperature from 380 to 500 K, with a heating ramp of 10 K·min^−1^, was applied. The solid samples in equilibrium with the saturated solution were dried at room temperature for 48 h under a continuous stream of dry air [56].

## 5. Conclusions

The dissolution of SMR in solvent mixtures is an endothermic process. The results show a possible relationship between the acidic character of the solvents and the solubility of SMR, with a higher affinity for media with a basic character. As far as the thermodynamic functions are concerned, the process is driven by the solution entropy; the mixing process is also favored by entropy, but the values obtained are within the range of error, so they are not conclusive. Regarding the enthalpic–entropic compensation, the linear relationship shows a strongly compensated process. Finally, the experimental solubility data were correlated with the van’t Hoff–Yalkowsky–Roseman model, with excellent results, highlighting that only 4 experimental data were used to calculate 189 data. Taking into account the works related to the solubility of SMR in MeCN + MeOH and MeCN-PrOH, the influence of the aliphatic chain of the alcohols can be observed in the solubility behavior of SMR. In MeCN + MeOH (one -${\mathrm{CH}}_{3}$ group), the solubility tendency of SMR seems to be more related to the solubility parameter of the cosolvent mixtures; in this cosolvent system, the maximum solubility is reached in a cosolvent mixture with a solubility parameter similar to that of SMR. In MeCN + EtOH (one -CH_2_- group and one -${\mathrm{CH}}_{3}$ group) and MeCN-PrOH (two -${\mathrm{CH}}_{2}$- groups and one -${\mathrm{CH}}_{3}$ group), the solubility tendency seems to be more related to the acidic/basic character of the solvents. Therefore, this work allows us to conclude that the change in the number of carbons of the aliphatic chain of the alcohol in the MeCN–alcohol cosolvent mixture induces changes in the mechanisms involved in the solubility of SMR, highlighting the difficulty of developing mathematical models that allow solubility to be predicted, since this property clearly does not follow a predictable pattern in this case.

## Figures and Tables

**Table 1:** Experimental solubility of SMR (3) ($10^{4}x_{3}$) in {MeCN (1) + EtOH (2)} mixtures expressed as mole fraction at several temperatures. Experimental pressure *p*: 0.096 MPa ^*a*^.

${w_{1}}^{b}$	Temperature (K) ^***c***^				
	**278.15**	**283.15**	**288.15**	**293.15**	**298.15**
0.00	1.360 ± 0.024	1.767 ± 0.015	2.2 ± 0.03	2.74 ± 0.06 ^*d*^	3.41 ± 0.10 ^*d*^
0.05	1.505 ± 0.024	1.94 ± 0.05	2.412 ± 0.026	3.01 ± 0.07	3.831 ± 0.032
0.10	1.69 ± 0.04	2.14 ± 0.06	2.66 ± 0.07	3.4 ± 0.05	4.19 ± 0.04
0.15	1.92 ± 0.016	2.386 ± 0.022	2.974 ± 0.021	3.69 ± 0.05	4.54 ± 0.06
0.20	2.13 ± 0.03	2.634 ± 0.023	3.30 ± 0.08	4.07 ± 0.09	4.93 ± 0.06
0.25	2.386 ± 0.008	2.99 ± 0.04	3.66 ± 0.09	4.6 ± 0.08	5.48 ± 0.11
0.30	2.66 ± 0.04	3.30 ± 0.06	4.02 ± 0.06	5.00 ± 0.08	5.89 ± 0.04
0.35	3.04 ± 0.05	3.69 ± 0.07	4.56 ± 0.09	5.53 ± 0.04	6.68 ± 0.07
0.40	3.41 ± 0.04	4.145 ± 0.033	4.96 ± 0.10	6.20 ± 0.05	7.33 ± 0.15
0.45	3.78 ± 0.08	4.61 ± 0.09	5.56 ± 0.07	6.709 ± 0.022	8.06 ± 0.15
0.50	4.28 ± 0.05	5.08 ± 0.06	6.08 ± 0.06	7.38 ± 0.05	8.69 ± 0.13
0.55	4.69 ± 0.008	5.63 ± 0.13	6.92 ± 0.02	8.19 ± 0.2	9.71 ± 0.11
0.60	5.26 ± 0.04	6.32 ± 0.11	7.567 ± 0.027	9.14 ± 0.19	10.81 ± 0.15
0.65	6.03 ± 0.06	7.06 ± 0.09	8.45 ± 0.25	9.95 ± 0.04	11.87 ± 0.30
0.70	6.62 ± 0.07	7.93 ± 0.10	9.28 ± 0.26	11.06 ± 0.22	13.12 ± 0.16
0.75	7.45 ± 0.16	8.74 ± 0.14	10.132 ± 0.03	12.14 ± 0.08	14.13 ± 0.24
0.80	8.30 ± 0.13	9.84 ± 0.19	11.62 ± 0.13	13.16 ± 0.07	15.6 ± 0.4
0.85	9.30 ± 0.11	10.84 ± 0.16	12.91 ± 0.26	15.07 ± 0.12	17.07 ± 0.28
0.90	10.59 ± 0.04	12.1 ± 0.22	14.03 ± 0.33	16.46 ± 0.16	19.0 ± 0.5
0.95	11.84 ± 0.13	13.58 ± 0.29	15.43 ± 0.12	18.37 ± 0.23	20.6 ± 0.5
1.00	13.09 ± 0.10 ^*e*^	15.10 ± 0.07 ^*e*^	17.40 ± 0.09 ^*e*^	20.09 ± 0.18 ^*e*^	22.86 ± 0.27 ^*e*^
${w_{1}}^{b}$	**Temperature (K)** ^***c***^				
	**303.15**	**308.15**	**313.15**	**318.15**	
0.00	4.09 ± 0.09 ^*d*^	4.95 ± 0.04 ^*d*^	6.36 ± 0.11 ^*d*^	7.49 ± 0.14	
0.05	4.45 ± 0.04	5.45 ± 0.06	7.06 ± 0.21	8.26 ± 0.17	
0.10	4.97 ± 0.1	5.92 ± 0.11	7.69 ± 0.14	9.08 ± 0.18	
0.15	5.38 ± 0.11	6.52 ± 0.07	8.49 ± 0.21	10.15 ± 0.19	
0.20	5.93 ± 0.06	7.38 ± 0.12	9.28 ± 0.25	11.11 ± 0.2	
0.25	6.61 ± 0.09	8.08 ± 0.22	10.23 ± 0.17	12.36 ± 0.31	
0.30	7.14 ± 0.13	8.81 ± 0.23	11.34 ± 0.31	13.9 ± 0.26	
0.35	8.00 ± 0.11	9.71 ± 0.09	12.4 ± 0.3	15.12 ± 0.26	
0.40	8.58 ± 0.10	10.62 ± 0.07	13.64 ± 0.22	16.9 ± 0.4	
0.45	9.67 ± 0.19	11.93 ± 0.18	15.0 ± 0.4	18.59 ± 0.29	
0.50	10.62 ± 0.03	12.9 ± 0.06	16.5 ± 0.4	20.5 ± 0.4	
0.55	11.57 ± 0.19	14.6 ± 0.4	18.17 ± 0.19	22.8 ± 0.7	
0.60	12.64 ± 0.27	15.8 ± 0.4	20.1 ± 0.3	25.3 ± 0.6	
0.65	13.92 ± 0.4	17.69 ± 0.2	21.7 ± 0.14	28.31 ± 0.35	
0.70	15.3 ± 0.27	19.05 ± 0.09	24.0 ± 0.5	31.5 ± 0.5	
0.75	16.7 ± 0.19	21.67 ± 0.31	27.0 ± 0.9	34.4 ± 0.7	
0.80	18.8 ± 0.24	23.87 ± 0.29	30.0 ± 0.5	37.8 ± 0.7	
0.85	20.56 ± 0.06	25.9 ± 0.4	32.2 ± 0.6	42.1 ± 1.2	
0.90	22.51 ± 0.32	28.6 ± 0.7	35.7 ± 0.8	46.7 ± 0.5	
0.95	24.88 ± 0.4	31.9 ± 0.4	39.5 ± 0.5	52.9 ± 0.4	
1.00	27.14 ± 0.14 ^*e*^	34.7 ± 0.5 ^*e*^	43.9 ± 0.32 ^*e*^	57.2 ± 0.9 ^*e*^	

^*a*^ Standard uncertainty in pressure *u(p)* = 0.001 MPa; ^*b*^ $w_{1}$ is the mass fraction of MeCN (1) in the MeCN (1) + EtOH (2) mixtures free of SMR (3); ^*c*^ standard uncertainty in temperature is *u(T)* = 0.05 K; ^*d*^ from Delgado and Martínez [25]; ^*e*^ from Blanco-Márquez et al. [29].

**Table 2:** Thermophysical properties of SMR obtained by DSC.

Sample	Enthalpy of Melting, $\Delta_{m}H$${\mathrm{/kJ\cdot mol}}^{-1}$	Melting Point $T_{m}$ (K)	Ref.
Original sample ^a^	41.5 ± 0.5	508.1 ± 0.5	
	41.3 ± 0.5	508.5 ± 0.5	Ortiz et al. [21]
	31.6	515.2	Sunwoo and Eisen [34]
	24.75	509.3–510.3	Lee et al. [35]
	41.3	508.5	Martínez and Gómez [36]
	41.3 ± 1.0	508.5	Delgado and Martínez [25]
		508.9	Blanco-Márquez et al. [29]
		506.4	Khattab [37]
		508.95	Delombaerde [38]
		510.66	Aloisio et al. [39]
		508.5	Cárdenas-Torres et al. [22]
		508.5	Vargas-Santana et al. [30]
EtOH	41.6 ± 0.5	510.5 ± 0.5	
$w_{0.50}$	40.5 ± 0.5	509.2 ± 0.5	
Acetonitrile	41.2 ± 0.5	510.2 ± 0.5	
Acetonitrile	40.9 ± 0.5	509.1 ± 0.5	Ortiz et al. [21]

^a^ High-purity commercial standard.

**Table 4:** Thermodynamic functions relative to mixing processes of SMR (3) in {MeCN (1) + EtOH (2)} cosolvent mixtures at $T_{\mathrm{hm}}=297.6$ K ^a^.

$w_{1}$ ^b^	$\Delta_{\mathrm{mix}}G^{\circ }$	$\Delta_{\mathrm{mix}}H^{\circ }$	$\Delta_{\mathrm{mix}}S^{\circ }$	$T\Delta_{\mathrm{mix}}S^{\circ }$
	(kJ·mol^−1^)	(kJ·mol^−1^)	(J·mol^−1^·K^−1^)	(kJ·mol^−1^)
0.00	6.9 ± 0.14	7.00 ± 0.27	0.3 ± 0.5	0.01 ± 0.14
0.05	6.7 ± 0.3	7.1 ± 0.3	1.3 ± 0.8	0.40 ± 0.25
0.10	6.4 ± 0.4	6.6 ± 0.3	0.8 ± 0.9	0.23 ± 0.27
0.15	6.2 ± 0.3	6.3 ± 0.4	0.4 ± 0.7	0.13 ± 0.22
0.20	5.92 ± 0.33	6.21 ± 0.33	1.0 ± 0.8	0.28 ± 0.24
0.25	5.7 ± 0.3	5.8 ± 0.4	0.5 ± 0.8	0.15 ± 0.25
0.30	5.4 ± 0.3	5.8 ± 0.5	1.4 ± 1	0.40 ± 0.29
0.35	5.15 ± 0.29	5.1 ± 0.4	−0.1 ± 0.8	−0.03 ± 0.25
0.40	4.91 ± 0.26	4.8 ± 0.5	−0.4 ± 0.9	−0.12 ± 0.26
0.45	4.65 ± 0.30	4.7 ± 0.5	0.3 ± 0.9	0.08 ± 0.27
0.50	4.42 ± 0.21	4.4 ± 0.6	0.0 ± 0.9	0.01 ± 0.26
0.55	4.16 ± 0.28	4.4 ± 0.6	0.9 ± 1	0.27 ± 0.3
0.60	3.91 ± 0.28	4.1 ± 0.6	0.7 ± 1.1	0.21 ± 0.32
0.65	3.65 ± 0.26	3.6 ± 0.7	0.0 ± 1.1	0.00 ± 0.33
0.70	3.41 ± 0.26	3.5 ± 0.7	0.4 ± 1.2	0.1 ± 0.4
0.75	3.16 ± 0.26	3.6 ± 0.8	1.3 ± 1.3	0.4 ± 0.4
0.80	2.9 ± 0.24	3.2 ± 0.8	1.1 ± 1.3	0.3 ± 0.4
0.85	2.66 ± 0.24	2.7 ± 0.8	0.3 ± 1.3	0.1 ± 0.4
0.90	2.4 ± 0.27	2.5 ± 0.9	0.4 ± 1.4	0.1 ± 0.4
0.95	2.14 ± 0.22	2.5 ± 1.0	1.4 ± 1.5	0.4 ± 0.5
1.00	1.91 ± 0.05	2.0 ± 0.9	0.4 ± 1.4	0.1 ± 0.4

^a^ Average relative standard uncertainty in *w*_1_ is *u*_*r*_(*w*_1_) = 0.0008. Standard uncertainty in *T* is *u*(*T*) = 0.10 K. ^b^ *w*_1_ is the mass fraction of MeCN (1) in the {MeCN (1) + EtOH (2)} mixtures free of SMR (3).

**Table 5:** Source and purity of the compounds used in this research study.

Chemical Name	CAS ^***a***^	Purity in Mass Fraction	Analytic Technique ^*b*^
Sulfamerazine ^*c*^	127-79-7	>0.990	HPLC
Ethanol ^*c*^	64-17-5	0.998	GC
Acetonitrile ^*d*^	75-05-8	0.998	GC

^*a*^ Chemical Abstracts Service Registry Number. ^*b*^ HPLC is high-performance liquid chromatography; GC is gas chromatography. ^*c*^ Sigma-Aldrich, Burlington, MA, USA. ^*d*^ Supelco, Burlington, MA, USA.

## Data Availability

Data are contained within the article.
